# Clinical characteristics and outcomes of liver abscesses: a 12-year retrospective analysis from a tertiary care center in the United Arab Emirates

**DOI:** 10.3389/fmed.2025.1673473

**Published:** 2025-12-09

**Authors:** Adnan Agha, Mohammed Fareeduddin Farooqi, Maria Khan, Noria Ghulam Nabi, Amatur Rehman Siddiqua

**Affiliations:** 1 Department of Internal Medicine, College of Medicine and Health Sciences, United Arab Emirates University, Al Ain, United Arab Emirates; 2 Department of Medicine, Tawam Hospital, Al Ain, United Arab Emirates

**Keywords:** liver abscess, pyogenic liver abscess, amoebic liver abscess, *Klebsiella pneumoniae*, United Arab Emirates, mortality

## Abstract

**Background:**

Liver abscesses are a cause of morbidity worldwide and their outcomes can vary remarkably in various regions of the world. In the United Arab Emirates (UAE) the clinical picture of liver abscesses is not fully understood. We aimed to fill this gap by analyzing the clinical features, microbiology, and outcomes for patients with liver abscess over a 12-year period.

**Methods:**

We conducted a retrospective observational study of all adult patients (≥18 years) with confirmed diagnosis of liver abscess at Tawam Hospital, Al Ain, UAE, from January 2012 to January 2024. Electronic medical records were reviewed to extract demographic, clinical, laboratory, radiological, and microbiological data. Patients were then classified as either having pyogenic liver abscess (PLA) or amoebic liver abscess (ALA). Statistical analyses were performed to identify predictors of morbidity and mortality.

**Results:**

Of 158 patients screened, 79 had confirmed liver abscesses (71 pyogenic, 8 amoebic). The mean age was 56.8 ± 16.2 years, with male predominance (65.8%). The commonest clinical findings were abdominal pain (73.4%), fever (68.4%), and nausea/vomiting (45.6%). Diabetes mellitus was found in 35.4% of patients, while malignancy was identified in 30.4%. *Klebsiella pneumoniae* was the most frequently isolated organism (40.0% of positive pus cultures), followed by *Escherichia coli* (14.0%). However, *E. coli*, particularly ESBL-producing strains, showed disproportionately high mortality (80.0%) compared to *K. pneumoniae* (2.6% mortality). The overall mortality rate was 15.2% (12/79), with 83.3% (10/12) directly attributable to liver abscess complications. ICU admission was required in 20.3% of cases. In univariate analysis, predictors of mortality included age >65 years (OR 12.2, 95% CI 2.9–51.2, *p* < 0.001), serum albumin <30 g/L (OR 17.6, 95% CI 2.2–142.8, *p* < 0.001), and presence of multiple abscesses (OR 4.5, 95% CI 1.3–15.6, *p* = 0.02).

**Conclusion:**

The overwhelming majority of patients with liver abscesses at high-risk for complications respond favorably to antimicrobial therapy. However, the most striking finding was the unexpectedly observed mortality associated with *E. coli*, especially ESBL strains (80% mortality), something that underscores the need for rapid diagnosis and robust empiric coverage for resistant organisms in the severely ill. Our findings identify increased frequency of underlying malignancy which emerged as a major determinant of mortality, reinforcing the need for comprehensive cancer screening into the diagnostic workup of patients presenting with liver abscess.

## Introduction

Liver abscess is a localized collection of pus within the parenchyma of the liver which can be a consequence of infections (bacterial, parasitic, or fungal). Liver abscesses present a global health challenge, but their etiology, microbiology, and outcomes vary considerably across geographical regions (1, 2). Incidence rates are highest in East and Southeast Asia (17.6 per 100,000 in Taiwan) compared to Western countries (2.3 and 7 per 100,000 in the United States and Germany respectively), primarily due to differences in prevalence of pathogens, underlying biliary disease, and diabetes mellitus prevalence (3, 4). The epidemiology of liver abscesses has evolved over recent decades. Pyogenic liver abscesses (PLA) are the most common type in developed countries, typically arising from biliary tract infections, portal vein seeding, or direct extension from adjacent structures (1, 5). *Klebsiella pneumoniae* has emerged as a dominant pathogen, particularly in Southeast Asia where its hypervirulent strain has spread throughout Asia and globally, which can cause invasive liver abscess syndrome with metastatic complications (6). In contrast, amoebic liver abscesses (ALA) caused by *Entamoeba histolytica* remain prevalent in tropical and subtropical regions with poor sanitation-related health issues (7).

The United Arab Emirates (UAE) presents a unique epidemiological setting due to its diverse population, with expatriates comprising approximately 88% of residents (8). This demographic diversity, combined with rapid socioeconomic development and modern healthcare infrastructure, creates distinct patterns of infectious diseases. Despite this, data on liver abscesses in the UAE remain limited. The previous study from UAE by Mousa et al. (9) reported on 45 patients between 2012 and 2018, finding *Klebsiella pneumoniae* in 42.5% of cases and *Entamoeba histolytica* in 15% of cases, with an overall mortality of 7.5%. However, it is important to note that their proportion represents all cases, whereas culture yield and denominators used can significantly affect reported proportions; and this study did not examine the predictors of mortality or the role of underlying malignancy in liver abscesses. The current healthcare landscape in the UAE, with mandatory health insurance coverage and advanced diagnostic capabilities, provides an opportunity to comprehensively assess liver abscess epidemiology and outcomes.

Understanding the local epidemiology, microbiological patterns, and clinical outcomes of liver abscesses is crucial for optimizing management strategies and improving patient outcomes. This study aimed to assess the characteristics of patients with liver abscesses over a 12-year period and identify the predictors of morbidity and mortality, including Intensive care Unit (ICU) admissions and readmission rates in UAE population.

## Methods

### Study design and setting

We conducted a retrospective electronic medical chart review at Tawam Hospital, a tertiary care academic hospital in Al Ain, Abu Dhabi, UAE. The hospital serves as a regional referral center for approximately 750,000 residents in the eastern region of Abu Dhabi. The study was approved by the Institutional Review Board (IRB), SEHA Research Ethics Committee, with approval Number HREC SEHA-IRB-787 approved in June 2024. The study was in accordance with the ethical standards of Declaration of Helsinki and need for Informed consent was waived by the IRB due to the retrospective nature of the study with and the collection of de-identified data only.

### Patient selection

**Figure 1:**
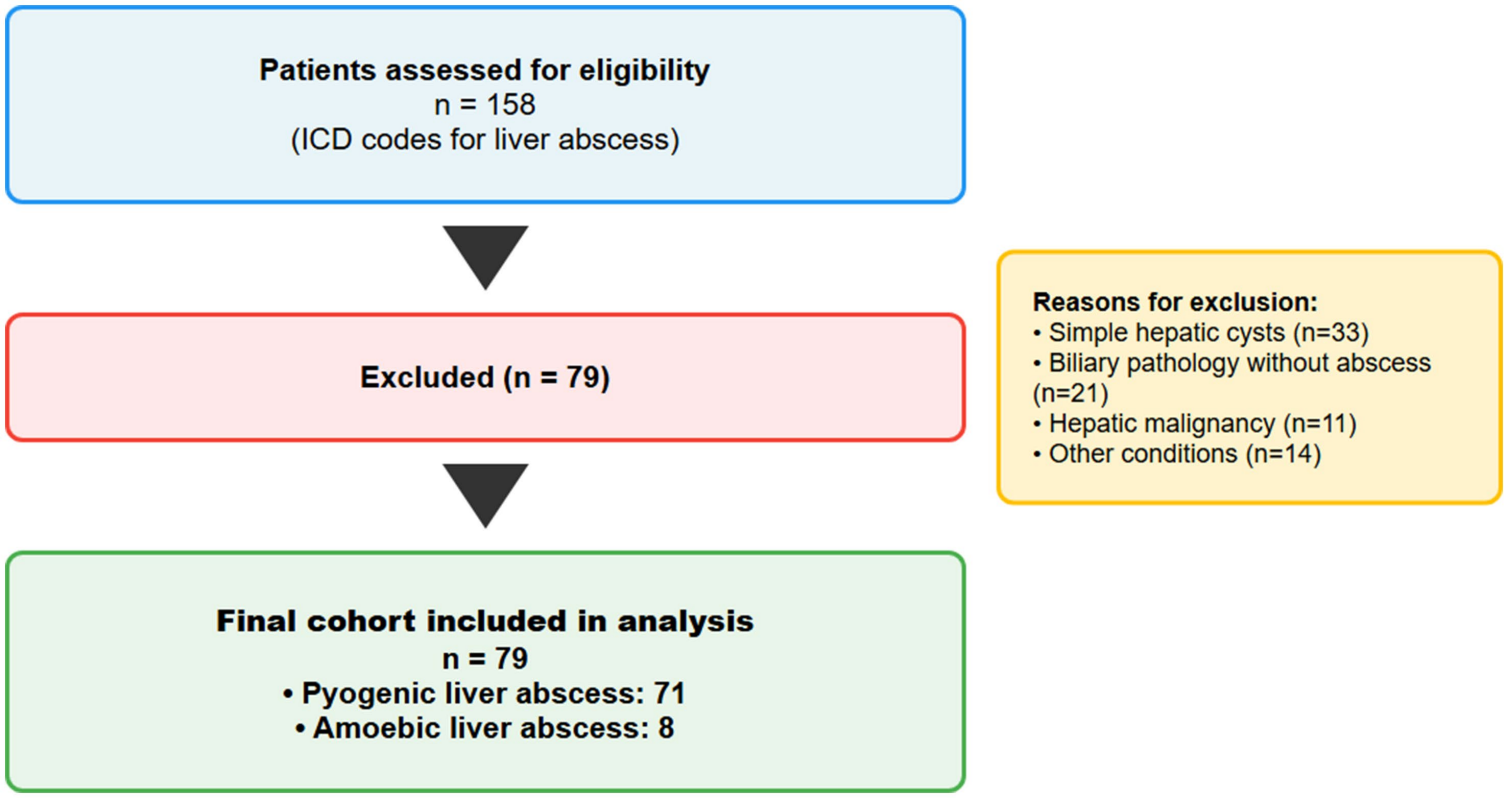
STROBE flow diagram of patient selection. Flow diagram illustrating the selection process for the study cohort according to STROBE (Strengthening the Reporting of Observational Studies in Epidemiology) guidelines. Of 158 patients initially identified through International Classification of Diseases (ICD) codes for liver abscess between January 2012 and January 2024, detailed chart review confirmed liver abscess diagnosis in 79 patients (50.0%). Excluded patients (*n* = 79) had alternative diagnoses including simple hepatic cysts (*n* = 33), biliary pathology without abscess formation (*n* = 21), hepatic malignancy initially suspected to be abscess (*n* = 11), or other non-abscess conditions (*n* = 14). The final cohort comprised 71 patients with pyogenic liver abscess (89.9%) and 8 patients with amoebic liver abscess (10.1%).

**Figure 2:**
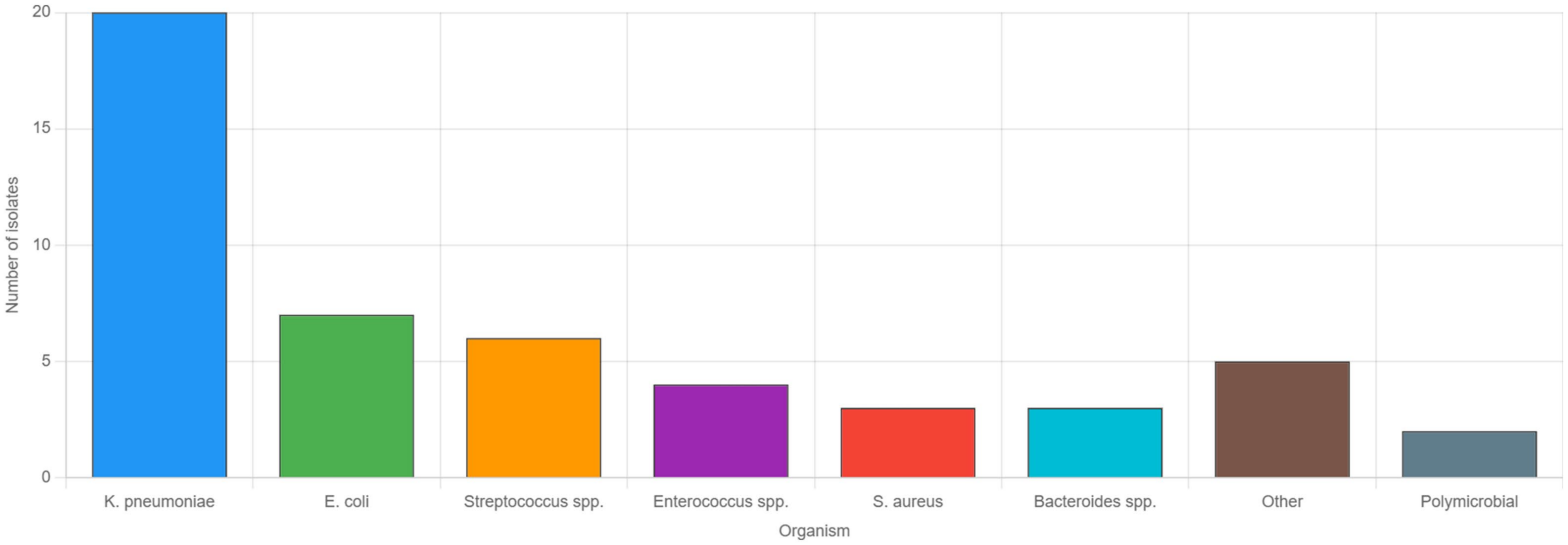
Microbiological profile of pyogenic liver abscesses. Bar graph showing the distribution of causative organisms identified from pus cultures in patients with pyogenic liver abscesses who had positive culture results (*n* = 50). *Klebsiella pneumoniae* was the predominant pathogen, isolated in 20 cases (40.0%), followed by *Escherichia coli* in 7 cases (14.0%), and *Streptococcus* species in 6 cases (12.0%). *Enterococcus species* were identified in 4 cases (8.0%), while *Staphylococcus aureus* and *Bacteroides species* were each found in 3 cases (6.0%). Other organisms, including less common gram-negative and gram-positive bacteria, accounted for five cases (10.0%), and polymicrobial infections were documented in two cases (4.0%). These findings reflect the microbiological spectrum of community-acquired pyogenic liver abscesses in the United Arab Emirates over the 12-year study period.

**Figure 3:**
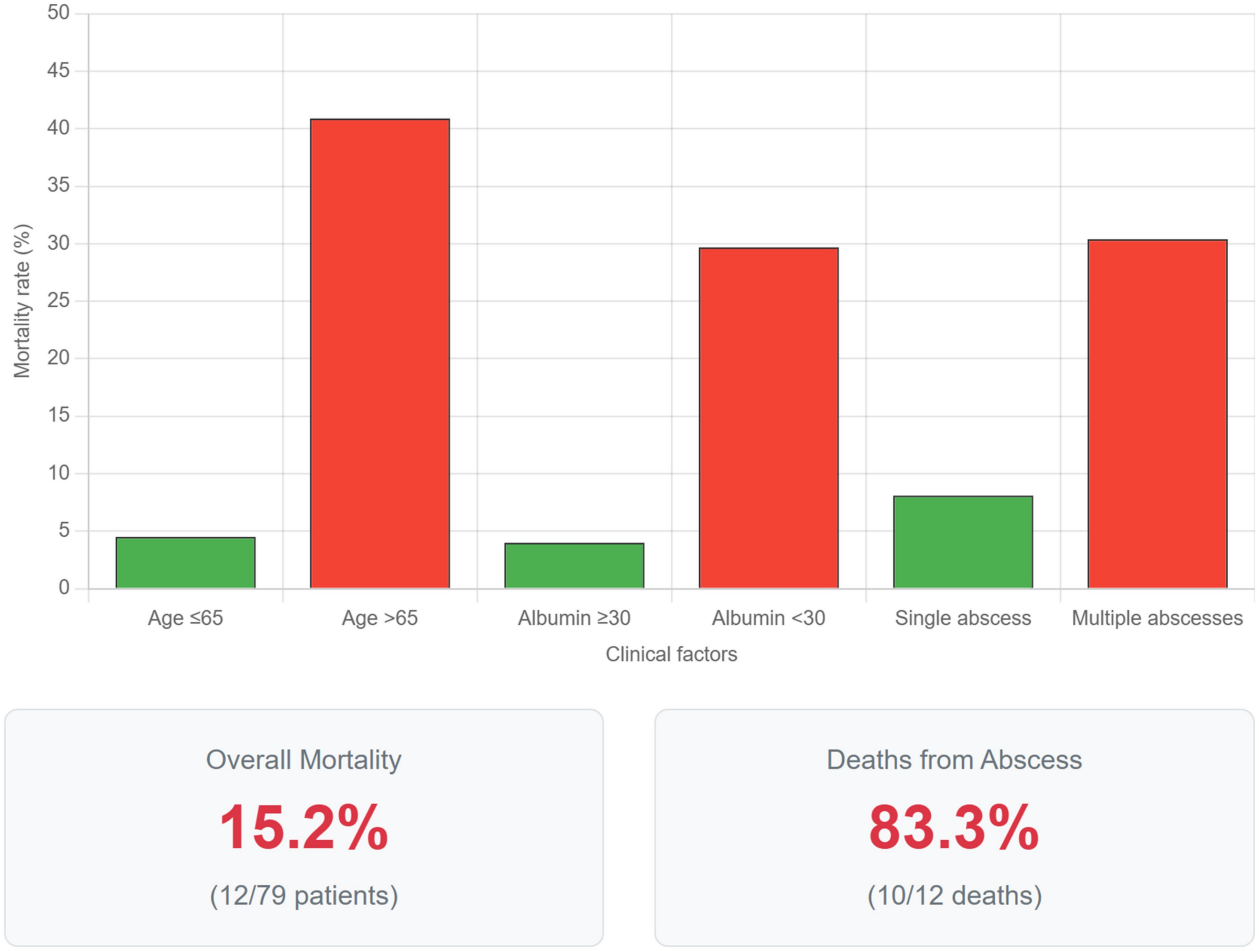
Factors associated with mortality in liver abscess patients. Comparative bar graph illustrating mortality rates stratified by key clinical factors identified as significant predictors in univariate analysis. Patients aged >65 years demonstrated a mortality rate of 40.9% compared to 4.5% in those ≤65 years (*p* < 0.001). Hypoalbuminemia (serum albumin <30 g/L) was associated with 29.7% mortality versus 4.0% in patients with normal albumin levels (*p* < 0.001). The presence of multiple abscesses resulted in 30.4% mortality compared to 8.1% with solitary abscesses (*p* = 0.02). Error bars represent 95% confidence intervals. The lower panel displays overall mortality statistics: 15.2% (12/79) overall mortality rate, with 83.3% (10/12) of deaths directly attributable to liver abscess complications. These findings emphasize the importance of age, nutritional status, and abscess burden in determining prognosis.

All adult patients (≥18 years) admitted with a primary diagnosis of liver abscess between January 1, 2012, and January 1, 2024, were identified using International Classification of Diseases (ICD) codes. For patients admitted before 2017, ICD-9 code 572.0 was used; for admissions from 2017 onwards, ICD-10 code K75.0 was used (10, 11). Electronic medical records were reviewed in detail to confirm the diagnosis of liver abscess based on clinical presentation, imaging findings, and microbiological confirmation where available (Figures 1–3).

### Data collection

The data were extracted from the electronic medical record system using a standardized data collection form. Variables collected included demographics like age, sex, nationality, insurance status; clinical data like signs and symptoms, vital signs, comorbidities; laboratory data like complete blood count, inflammatory markers etc.; and radiological findings like ultrasound imaging for abscess size, number, location and presence of gas formation. Microbiological data, including the results of blood and liver abscess pus cultures, were extracted from the electronic medical record. In accordance with institutional and international standards, blood cultures were processed using an automated continuous-monitoring system. Upon signalling positive, a Gram stain was performed directly from the positive broth for rapid preliminary reporting, and the broth was sub-cultured onto a panel of solid media including Blood Agar, MacConkey Agar, and Chocolate Agar. Pus specimens obtained via percutaneous drainage were directly Gram-stained and inoculated onto a similar panel of media, supplemented with appropriate anaerobic media and an enrichment broth. All solid media were incubated at 35–37 °C in the appropriate atmospheric conditions (aerobic or anaerobic) for up to 72 h. Subsequent bacterial identification to the species level and comprehensive antimicrobial susceptibility testing were performed using the Vitek 2 automated system (bioMérieux, Marcy-l’Étoile, France). This platform has been the established standard of practice at the institution for the entirety of the study period, having been implemented in 2006.^22^ All laboratory procedures were conducted in adherence to Clinical and Laboratory Standards Institute (CLSI) guidelines.

### Definitions

- Pyogenic liver abscess (PLA): Bacterial etiology confirmed by positive cultures or clinical response to antibacterial therapy
- Amoebic liver abscess (ALA): Positive *Entamoeba histolytica* serology or characteristic anchovy paste aspirate with response to metronidazole

### Statistical analysis

Data were analyzed using SPSS version 29.0 (IBM Corp., Armonk, NY). Continuous variables were expressed as mean ± standard deviation (SD) for normally distributed data or median with interquartile range (IQR) for non-normally distributed data. Categorical variables were expressed as frequencies and percentages. Comparisons between groups were performed using Student’s t-test or Mann–Whitney U test for continuous variables and chi-square or Fisher’s exact test for categorical variables. Univariate logistic regression was performed to identify predictors of mortality. Variables with *p* < 0.10 in univariate analysis were considered for multivariate analysis; however, due to the limited number of events (*n* = 9), multivariate modeling was not pursued to avoid overfitting. A two-tailed *p*-value <0.05 was considered statistically significant.

## Results

During the 12-year study period, 158 patients were initially identified with ICD codes for liver abscess. After detailed chart review, 79 (50.0%) had confirmed liver abscesses, while the remainder had alternative diagnoses including simple hepatic cysts (*n* = 33), biliary pathology without abscess (*n* = 21), hepatic malignancy (*n* = 11), or other conditions (*n* = 14).

Among the 79 patients with confirmed liver abscesses, 71 (89.9%) had pyogenic liver abscesses and 8 (10.1%) had amoebic liver abscesses. The demographic and clinical characteristics are summarized in Table 1.

**Table 1:** Demographic and clinical characteristics of patients with liver abscesses (*n* = 79).

Characteristic	Total (*n* = 79)	PLA (*n* = 71)	ALA (*n* = 8)	*p*-value
Age, mean ± SD (years)	56.8 ± 16.2	57.6 ± 16.0	49.6 ± 16.8	0.18
Male sex, *n* (%)	52 (65.8)	45 (63.4)	7 (87.5)	0.25
Nationality, *n* (%)				0.38
- Emirati	31 (39.2)	29 (40.8)	2 (25.0)	
- South Asian	28 (35.4)	23 (32.4)	5 (62.5)	
- Arab (non-Emirati)	15 (19.0)	14 (19.7)	1 (12.5)	
- Other	5 (6.3)	5 (7.0)	0 (0)	
Presenting symptoms, *n* (%)				
- Abdominal pain	58 (73.4)	51 (71.8)	7 (87.5)	0.44
- Fever	54 (68.4)	47 (66.2)	7 (87.5)	0.42
- Nausea/vomiting	36 (45.6)	32 (45.1)	4 (50.0)	0.99
- Jaundice	22 (27.8)	21 (29.6)	1 (12.5)	0.43
Comorbidities, *n* (%)				
- Diabetes mellitus	28 (35.4)	26 (36.6)	2 (25.0)	0.70
- Hypertension	25 (31.6)	23 (32.4)	2 (25.0)	0.99
- Malignancy	24 (30.4)	23 (32.4)	1 (12.5)	0.41
- Prior biliary surgery	12 (15.2)	11 (15.5)	1 (12.5)	0.99

Laboratory parameters at admission are shown in Table 2. Patients demonstrated leukocytosis, elevated inflammatory markers, and abnormal liver function tests in the majority of cases.

**Table 2:** Laboratory findings at admission for patients with liver abscess (*n* = 79).

Parameter	Mean ± SD or Median (IQR)	Reference range
WBC (× 10^9^/L)	13.8 ± 6.2	4.0–11.0
Neutrophils (× 10^9^/L)	10.9 ± 5.8	2.0–7.5
Hemoglobin (g/L)	118 ± 22	120–150
Platelets (× 10^9^/L)	285 ± 142	150–400
CRP (mg/L)	186 (124–268)	<5
ALT (U/L)	58 (35–92)	10–40
AST (U/L)	62 (38–98)	10–40
ALP (U/L)	145 (98–218)	40–130
Total bilirubin (μmol/L)	31 (18–58)	5–21
Albumin (g/L)	30.8 ± 7.2	35–50
INR	1.2 ± 0.3	0.8–1.2

Abdominal ultrasonography was performed in all patients as the initial imaging modality. CT was performed in 42 (53.2%) cases for further characterization. The imaging characteristics are summarized in Table 3.

**Table 3:** Radiological characteristics of liver abscesses (*n* = 79).

Characteristic	*n* (%)
Abscess location	
- Right lobe only	48 (60.8)
- Left lobe only	18 (22.8)
- Bilateral	13 (16.5)
Number of abscesses	
- Solitary	56 (70.9)
- Multiple (≥2)	23 (29.1)
Size of largest abscess	
- < 5 cm	23 (29.1)
- 5–10 cm	42 (53.2)
- > 10 cm	14 (17.7)
Gas-forming abscess	8 (10.1)

To facilitate the comparison with previous studies, we analyzed *Klebsiella pneumoniae* proportions using different denominators. *K. pneumoniae* was isolated in 38/158 (24.1%) of all liver abscess cases, 38/66 (57.6%) of culture-positive pyogenic abscesses, and 20/50 (40.0%) of positive pus cultures. The difference from the 42.5% reported by Mousa et al. (9) for all cases likely reflects our higher proportion of culture-negative cases and larger sample size, though the dominance of *K. pneumoniae* among pyogenic cases remains consistent. Blood cultures were obtained in 68 (86.1%) patients, with positive results in 26 (38.2%). Pus cultures were obtained from 62 patients who underwent drainage, yielding positive results in 50 (80.6%). The microbiological profiles are detailed in Table 4.

**Table 4:** Microbiological findings in pyogenic liver abscesses (*n* = 71).

Organism	Pus culture (*n* = 50 positive)	Blood culture (*n* = 26 positive)
*Klebsiella pneumoniae*	20 (40.0%)	10 (38.5%)
*Escherichia coli*	7 (14.0%)	5 (19.2%)
Streptococcus spp.	6 (12.0%)	4 (15.4%)
Enterococcus spp.	4 (8.0%)	2 (7.7%)
*Staphylococcus aureus*	3 (6.0%)	2 (7.7%)
Bacteroides spp.	3 (6.0%)	1 (3.8%)
Other organisms	5 (10.0%)	1 (3.8%)
Polymicrobial	2 (4.0%)	1 (3.8%)

Among the 65 patients who underwent percutaneous drainage, successful resolution was achieved in 58 (89.2%) with single drainage, while 7 (10.8%) required repeat procedures. Patients requiring repeat drainage had larger abscesses (mean 9.2 cm vs. 6.8 cm, *p* = 0.03) and were more likely to have gas-forming abscesses (42.9% vs. 6.9%, *p* = 0.02). Culture positivity was higher in the percutaneous drainage group compared to those managed conservatively (80.6% vs. 37.5%, *p* = 0.01).

Among the 26 patients with positive blood cultures, 22 had concordant organisms in pus cultures. The 4 discordant cases included: 2 patients with *Streptococcus anginosus* in pus but *Escherichia coli* in blood, 1 patient with polymicrobial pus culture (*Bacteroides* plus *Enterococcus*) but only *Bacteroides* in blood, and 1 patient with *K. pneumoniae* in pus but *Staphylococcus aureus* in blood (likely representing concurrent bacteremia from another source). In all discordant cases, antimicrobial therapy was adjusted to cover all identified organisms, typically with carbapenem plus metronidazole.

Management strategies and clinical outcomes are summarized in Table 5. Percutaneous drainage was performed in 65 (82.3%) patients, with the remainder managed with antibiotics alone or surgical drainage.

**Table 5:** Management and outcomes.

Variable	*n* (%) or Median (IQR)
Drainage procedure	
- Percutaneous drainage	65 (82.3)
- Surgical drainage	6 (7.6)
- Conservative management	8 (10.1)
Initial antibiotic therapy	
- Third-generation cephalosporin + metronidazole	42 (53.2)
- Carbapenem ± metronidazole	18 (22.8)
- Piperacillin-tazobactam	12 (15.2)
- Other	7 (8.9)
Duration of IV antibiotics (days)	14 (10–21)
Total antibiotic duration (days)	42 (28–56)
Clinical outcomes	
- Complete resolution	62 (78.5)
- Residual collection requiring repeat drainage	5 (6.3)
- Death	12 (15.2)
Length of hospital stay (days)	12 (8–19)
ICU admission	16 (20.3)
30-day readmission	8 (10.1)

### Mortality analysis

Twelve patients (15.2%) died during the hospitalization. Ten deaths (83.3%) were directly attributable to liver abscess complications (septic shock, multi-organ failure), while two deaths were due to underlying malignancy with the abscess as a contributory factor. Univariate analysis identified several factors associated with mortality as illustrated in Table 6. Due to the limited number of events (*n* = 12), multivariate analysis was not performed to avoid overfitting and unreliable estimates.

**Table 6:** Univariate analysis of factors associated with mortality.

Variable	Survivors (*n* = 67)	Non-survivors (*n* = 12)	OR (95% CI)	*p*-value
Age >65 years	13 (19.4%)	9 (75.0%)	12.2 (2.9–51.2)	<0.001
Male sex	42 (62.7%)	10 (83.3%)	3.0 (0.6–14.8)	0.19
Diabetes mellitus	22 (32.8%)	6 (50.0%)	2.0 (0.6–7.0)	0.26
Albumin <30 g/L	26 (38.8%)	11 (91.7%)	17.6 (2.2–142.8)	<0.001
Multiple abscesses	16 (23.9%)	7 (58.3%)	4.5 (1.3–15.6)	0.02
Gas-forming abscess	5 (7.5%)	3 (25.0%)	4.1 (0.9–19.6)	0.07
*K. pneumoniae* infection	17 (25.4%)	3 (25.0%)	1.0 (0.2–4.0)	0.98

The distribution of causative organisms differed significantly between survivors and non-survivors. While *Klebsiella pneumoniae* was the predominant pathogen overall (38/66, 57.6% of culture-positive pyogenic cases), it was associated with only one death, yielding a mortality rate of 2.6% (1/38). In striking contrast, *Escherichia coli* demonstrated disproportionately high mortality: Extended-spectrum Beta-lactamase (ESBL) producing *E. coli* had an 80.0% mortality rate (4/5 cases), while non-ESBL *E. coli* had a 50.0% mortality rate (2/4 cases). Among the 11 abscess-related deaths, *E. coli* (ESBL and non-ESBL combined) accounted for 4 cases (36.4%), despite representing only 13.6% (9/66) of culture-positive pyogenic abscesses. Mixed organisms and other Enterobacteriaceae (Enterobacter, Citrobacter) were also overrepresented in fatal cases. Table 7 illustrates the distribution of causative organisms in detail.

**Table 7:** Microbiological distribution and associated mortality in patients with liver abscesses.

Organism	Total cases	Deaths	Mortality rate	% of all deaths
*Klebsiella pneumoniae*	38	1	2.6%	3.6%
*E. coli* ESBL	5	4	80.0%	14.3%
*E. coli* (non-ESBL)	4	2	50.0%	7.1%
Mixed organisms	3	2	66.7%	7.1%
Enterobacter spp.	2	2	100%	7.1%
Citrobacter spp.	2	2	100%	7.1%
Other organisms	12	1	8.3%	3.6%
No growth/Not done	92	14	15.2%	50.0%

Mortality rates calculated as number of deaths divided by total cases for each organism. ESBL, extended-spectrum beta-lactamase.

## Discussion

This 12-year retrospective study provides comprehensive data on the clinical characteristics and outcomes of liver abscesses in the UAE, revealing important insights into the evolving epidemiology and prognostic factors in this unique demographic setting. The predominance of pyogenic liver abscesses (89.9%) over amoebic liver abscesses (10.1%) in our cohort represents a significant epidemiological shift in the Middle East region. While this distribution still shows a higher ALA prevalence than Western countries where ALA comprises less than 10% of cases, it represents a substantial reduction from neighboring countries where ALA traditionally accounts for 15–25% of cases (12–15). This shift likely reflects improvements in public health infrastructure, water sanitation, and food safety regulations in the UAE.

### Microbiological patterns

*Klebsiella pneumoniae* (*K. pneumoniae*) emerged as the dominant pathogen in our study (40.0% of positive pus cultures), consistent with the “Asian phenomenon” of hypervirulent strains that have spread beyond East Asia (6, 16). This prevalence is higher than traditionally reported in the Middle East but aligns with recent regional data showing increasing *K. pneumoniae* dominance (17, 18). The relatively low extended-spectrum Beta-lactamase (ESBL) prevalence (10.0%) among *K. pneumoniae* isolates from our liver abscess cohort contrasts with the overall ESBL prevalence of 23–28% reported in UAE community-acquired infections (19). This difference may reflect the distinct pathogenesis of hypervirulent *K. pneumoniae* causing liver abscesses, which typically lacks ESBL genes due to incompatible plasmids, as reported in recent studies, possibly reflecting the community-acquired nature of most cases (20).

Perhaps our most clinically significant finding is the starkly divergent mortality risk seen between the pathogens. Despite *K. pneumoniae* being the most prevalent organism, it demonstrated remarkably low mortality (2.6%), suggesting that current antimicrobial protocols are effective against most *K. pneumoniae* strains in our setting. Conversely, the alarmingly high mortality associated with *E. coli*, especially ESBL-producing strains (80% mortality), highlights these as high-risk pathogens requiring aggressive management. This finding is in complete contrast with some Asian studies where hypervirulent *K. pneumoniae* typically drives mortality (6). Instead our data aligns more closely with the Western studies where resistant Gram-negative organisms are associated with poor outcomes (21). The association may reflect both antimicrobial resistance and host factors, as *E. coli* liver abscesses often occur in patients with biliary pathology or malignancy. These findings suggest that initial empiric therapy should ensure adequate coverage for ESBL-producing organisms in critically ill patients, and rapid organism identification with susceptibility testing is crucial for risk stratification.

The high culture positivity rate from pus samples (80.6%) compared to blood cultures (38.2%) emphasizes the importance of obtaining direct abscess samples when feasible. Among patients with positive cultures from both sites, the concordance in organism identification supports the reliability of blood culture results when abscess drainage is not immediately possible.

### Morbidity and mortality

Our mortality rate of 15.2% is substantially higher than the 5–8% reported from Western countries and at the upper end of recent Middle Eastern data showing mortality rates of 8–16% (15, 22). The finding that 83.3% of deaths were directly attributable to abscess complications underscores the severity of this condition despite modern management approaches. The identification of advanced age, hypoalbuminemia, and multiple abscesses as mortality predictors aligns with international literature (23). The particularly strong association with hypoalbuminemia (OR 17.6) likely reflects both malnutrition and the severity of the inflammatory response. Notably, the high prevalence of underlying malignancy (30.4%) in our cohort, substantially higher than the 5–10% typically reported (24), may contribute to the elevated mortality rate. This finding suggests that liver abscesses in our population often occur in the context of significant comorbidity, requiring particularly vigilant management.

### Treatment

The high success rate of percutaneous drainage (82.3% of patients) with relatively few requiring surgical intervention supports current guidelines recommending this approach as first-line therapy (25, 26). The median duration of intravenous antibiotics (14 days) and total therapy (42 days) aligns with recent evidence suggesting that shorter courses may be effective in selected patients with good source control (27, 28).

### Study strengths and limitations

Strengths of this study include the 12-year study period, comprehensive data collection including microbiological and outcome data, and transparent reporting of the cohort derivation process. The study provides valuable epidemiological data from an understudied region with unique demographic characteristics.

Several limitations merit consideration. The retrospective design of our study introduces a potential selection as well as information bias. The single-center nature may limit generalizability to other UAE hospitals. The relatively small number of mortality events (*n* = 12) precluded any multivariate model testing; hence our analysis should only be considered as exploratory. The higher proportion of culture-negative cases (58.2%) may affect the generalizability of our microbiological findings, though this reflects real-world clinical practice where cultures are often obtained after antibiotic initiation. Our retrospective data abstraction unfortunately did not allow for the systematic collection of data on the hypermucoviscous phenotype of *K. pneumoniae* isolates and in hindsight this information would have provided deeper insight into the prevalence of hypervirulent strains within our cohort. Future prospective studies are warranted to fully characterize the molecular epidemiology and virulence factors of *K. pneumoniae* causing pyogenic liver abscesses in the UAE.

### Conclusion

This study reveals three main findings that are pertinent to the liver abscesses in the UAE including: a shift toward pyogenic etiology with *K. pneumoniae* predominance, an unexpectedly high prevalence of underlying malignancy, and identification of hypoalbuminemia as a predictor of mortality, which emphasizes the importance of nutritional assessment in these patients. These findings suggest that liver abscess management in the UAE should incorporate routine cancer screening and nutritional support alongside antimicrobial therapy. These findings may help guide empirical therapy decisions and risk stratification in similar settings.

## Data Availability

The raw data supporting the conclusions of this article will be made available by the authors, without undue reservation.
